# Supplementary material for: Genome-Wide Identification of Genes Encoding for Rho-Related Proteins in ‘Duli’ Pear (Pyrus betulifolia Bunge) and Their Expression Analysis in Response to Abiotic Stress
Source: Plants (Basel). 2022 Jun 19;11(12):1608. doi: 10.3390/plants11121608 (PMC9230837; doi:10.3390/plants11121608)
Supplement: Supplementary file 1 [file plants-11-01608-s001.zip › TableS1.pdf]

Table S1 The Sequence of Motif

Motif name	Sequence
Motif1	TCMLISYTSNTFPTDYVPTVFDNFSANVVVDGSTVNLGLWDTAGQEDYNR
Motif2	HPGAVPITTAQGEELRKLIGAPAYIECSSKTQQNVKAVFDAAIKVVLQPP
Motif3	LRPLSYRGADVFLLAFSLISRASYENVAKKWIPELRHYAPGVPIILVGTK
Motif4	SRFIKCVTVGDGAVG
Motif5	DLREDKQFLAD
Motif6	QKKKKRKAQKACSIL
Motif7	ILCGRRLVCLK
