# Supplementary material for: Genome-Wide Identification of Genes Encoding for Rho-Related Proteins in ‘Duli’ Pear (Pyrus betulifolia Bunge) and Their Expression Analysis in Response to Abiotic Stress
Source: Plants (Basel). 2022 Jun 19;11(12):1608. doi: 10.3390/plants11121608 (PMC9230837; doi:10.3390/plants11121608)
Supplement: Supplementary file 1 [file plants-11-01608-s001.zip › TableS2.pdf]

Table S2 Ka/Ks of PbROP genes between '*Duli*' and Arabidopsis

Gene in *P. betulifolia*	Gene in *A.thaliana*	Ka	Ks	Ka/Ks
PbROP1	*AtROP1*	0.022	1.79	0.012496287
PbROP1	*AtROP3*	0.015	2.24	0.007007816
PbROP1	*AtROP5*	0.022	1.95	0.011516357
PbROP2	*AtROP2*	0.060	NaN	NaN
PbROP2	*AtROP4*	0.052	NaN	NaN
PbROP3	*AtROP6*	0.041	1.38	0.03018674
PbROP4	*AtROP2*	0.066	1.83	0.036246344
PbROP4	*AtROP4*	0.059	1.97	0.030083471
PbROP5	*AtROP6*	0.039	1.18	0.033552258
PbROP6	*AtROP6*	0.039	1.22	0.032575434
PbROP7	*AtROP9*	0.054	2.26	0.024046326
PbROP8	*AtROP8*	0.160	1.41	0.114372345
PbROP9	*AtROP9*	0.046	1.86	0.024932052
PbROP10	*AtROP10*	0.088	1.59	0.055382419
PbROP10	*AtROP11*	0.104	1.71	0.060822966
PbROP11	*AtROP10*	0.092	2.42	0.038045921
PbROP11	*AtROP11*	0.114	1.74	0.065533569
PbROP12	*AtROP8*	0.163	1.64	0.099379527
