# Supplementary material for: Genome-Wide Identification of Genes Encoding for Rho-Related Proteins in ‘Duli’ Pear (Pyrus betulifolia Bunge) and Their Expression Analysis in Response to Abiotic Stress
Source: Plants (Basel). 2022 Jun 19;11(12):1608. doi: 10.3390/plants11121608 (PMC9230837; doi:10.3390/plants11121608)
Supplement: Supplementary file 1 [file plants-11-01608-s001.zip › TableS3.pdf]

Table S3 Primer sequences used in *Pyrus betulaefolia* gene families

Primer name	Sequence 5'-3'
PbROP1F	CTACACCAGCAACACCTTC
PbROP1R	AGCACCACGATAACTCAA
PbROP2F	CTCCAGGTTCATAAAGTGC
PbROP2R	GATGGGAGCACTGTCAAC
PbROP3F	GATGTCTTCCTACTTGCGTTCT
PbROP3R	TTCTTCTGCTTCGGTGGC
PbROP4F	CTGTCAACCTGGCTCTATG
PbROP4R	AGCAAGTCCTCTGTCCCT
PbROP5F	CTCATAAGCAAGGCCAGCTATG
PbROP5R	TCCTTAGTTCCTCTCCCTG
PbROP6F	AATGTTGTTGTGGACGGG
PbROP6R	GCTAGCCTTGCTTATGAGAG
PbROP7F	TTGATGGGAACATAGTGAA
PbROP7R	CGATACTGCGATTAAGGT
PbROP8F	ATTTGAAGAAGCGAATAGG
PbROP8R	ATCCTCCAAAGTCCGAGA
PbROP9F	TGGCTGTGGATGGGAATA
PbROP9R	GGAGGATTACAGCAGGTT
PbROP10F	GTCTTTGTCTTGGCTTTCT
PbROP10R	TCACATTCTGTTGGGTTT
PbROP11F	GTGAACTTAGGCTTATGGG
PbROP11R	TCCTTCTGCTTCTGTGGC
PbROP12F	TGCCTTCTCATCTCCTAC
PbROP12R	TTCAAATCTTCACCCTGT
Pbβ-ActinF	TTGGTATGGGTCAGAAGG
Pbβ-ActinR	CTGTGAGCAGAACTGGGTG
CPbROP1F	ggggacaagtttgtacaaaaaagcaggctccaccatgagcgcgtccaggttcataaag
CPbROP1R	ggggaccactttgtacaagaaagctgggtTCACAATATGGAGCATGCCTTCTG
CPbROP2F	ggggacaagtttgtacaaaaaagcaggctccaccatgagcgcctccaggttcataaag
CPbROP2R	ggggaccactttgtacaagaaagctgggttcacaatatgtagcaagccctttgtc
CPbROP3F	ggggacaagtttgtacaaaaaagcaggctccaccatgagtgcctccaggttcataaag
CPbROP3R	ggggaccactttgtacaagaaagctgggtTCACAATATGGAGCAGGCCTTCTG
CPbROP9F	ggggacaagtttgtacaaaaaagcaggctccaccatgagtgcttcaaagttcattaaatgtg
CPbROP9R	ggggaccactttgtacaagaaagctgggtCTAAACATCACAGCCTCCACAGACAATG
