# Supplementary material for: Genome-Wide Identification of Genes Encoding for Rho-Related Proteins in ‘Duli’ Pear (Pyrus betulifolia Bunge) and Their Expression Analysis in Response to Abiotic Stress
Source: Plants (Basel). 2022 Jun 19;11(12):1608. doi: 10.3390/plants11121608 (PMC9230837; doi:10.3390/plants11121608)
Supplement: Supplementary file 1 [file plants-11-01608-s001.zip › TableS4.pdf]

Table S4 Synteny relationship between *ROP* gene in '*Duli*'and Arabidopsis

Pyrus betulifolia	ID number	*Arabidopsis thaliana*	ID number
PbROP1	Chr15.g02695.m2	AtROP4	AT1G75840
PbROP1	Chr15.g02695.m2	AtROP3	AT2G17800
PbROP1	Chr15.g02695.m2	AtROP1	AT3G51300
PbROP1	Chr15.g02695.m2	AtROP5	AT4G35950
PbROP2	Chr15.g04856.m1	AtROP4	AT1G75840
PbROP2	Chr15.g04856.m1	AtROP2	AT1G20090
PbROP2	Chr15.g04856.m1	AtROP3	AT2G17800
PbROP2	Chr15.g04856.m1	AtROP5	AT4G35950
PbROP2	Chr15.g04856.m1	AtROP6	AT4G35020
PbROP3	Chr10.g15874.m1	AtROP6	AT4G35020
PbROP4	Chr8.g53635.m1	AtROP4	AT1G75840
PbROP4	Chr8.g53635.m1	AtROP2	AT1G20090
PbROP4	Chr8.g53635.m1	AtROP5	AT4G35950
PbROP5	Chr5.g07968.m1	AtROP6	AT4G35020
PbROP10	Chr14.g49938.m2	AtROP10	AT3G48040
PbROP10	Chr14.g49938.m2	AtROP11	AT5G62880
PbROP11	Chr6.g51364.m1	AtROP10	AT3G48040
PbROP11	Chr6.g51364.m1	AtROP11	AT5G62880
PbROP12	Chr7.g33937.m1	AtROP8	AT2G44690
